# Development of sustainable biomass residues for biofuels applications

**DOI:** 10.1038/s41598-023-41446-1

**Published:** 2023-08-30

**Authors:** Mudasir Akbar Shah, Gasim Hayder, Rahul Kumar, Vimal Kumar, Tansir Ahamad, Md. Abul Kalam, Manzoore Elahi Mohammad Soudagar, Sathgatta Zaheeruddin Mohamed Shamshuddin, Nabisab Mujawar Mubarak

**Affiliations:** 1 Institute of Energy Infrastructure (IEI), Universiti Tenaga Nasional (UNITEN), 43000 Kajang, Malaysia; 2 Department of Chemical Engineering, Indian Institute of Technology Roorkee, Roorkee, Uttarakhand 247667 India; 3 Department of Chemistry, College of Science, King Saud University, Riyadh, Saudi Arabia; 4 School of Civil and Environmental Engineering, FEIT, University of Technology Sydney, NSW 2007, Australia; 5 Department of Mechanical Engineering and University Centre for Research and Development, Chandigarh University, Mohali, Punjab 140413 India; 6 Institute of Sustainable Energy, Universiti Tenaga Nasional, Jalan IKRAM-UNITEN, 43000, Kajang, Selangor, Malaysia; 7 Chemistry Research Laboratory, HMS Institute of Technology, Tumakuru, Karnataka 572104 India; 8 Petroleum and Chemical Engineering, Faculty of Engineering, Universiti Teknologi Brunei, Bandar Seri Begawan, BE1410 Brunei Darussalam; 9 Department of Biosciences, Saveetha School of Engineering, Saveetha Institute of Medical and Technical Sciences, Chennai, India

**Keywords:** Environmental sciences, Environmental social sciences, Energy science and technology

## Abstract

A comprehensive understanding of physiochemical properties, thermal degradation behavior and chemical composition is significant for biomass residues before their thermochemical conversion for energy production. In this investigation, teff straw (TS), coffee husk (CH), corn cob (CC), and sweet sorghum stalk (SSS) residues were characterized to assess their potential applications as value-added bioenergy and chemical products. The thermal degradation behavior of CC, CH, TS and SSS samples is calculated using four different heating rates. The activation energy values ranged from 81.919 to 262.238 and 85.737–212.349 kJ mol^−1^ and were generated by the KAS and FWO models and aided in understanding the biomass conversion process into bio-products. The cellulose, hemicellulose, and lignin contents of CC, CH, TS, and SSS were found to be in the ranges of 31.56–41.15%, 23.9–32.02%, and 19.85–25.07%, respectively. The calorific values of the residues ranged from 17.3 to 19.7 MJ/kg, comparable to crude biomass. Scanning electron micrographs revealed agglomerated, irregular, and rough textures, with parallel lines providing nutrient and water transport pathways in all biomass samples. Energy Dispersive X-ray spectra and X-ray diffraction analysis indicated the presence of high carbonaceous material and crystalline nature. FTIR analysis identified prominent band peaks at specific wave numbers. Based on these findings, it can be concluded that these residues hold potential as energy sources for various applications, such as the textile, plastics, paints, automobile, and food additive industries.

## Introduction

Due to the growing population and subsequent high energy demand and environmental concerns, government and non-government agencies demand higher environmental accountability for chemicals, fuels, and energy extraction. Biofuels and chemicals obtained from renewable sources are critical to solving global energy demand and reducing dependence on fossil fuels. Energy accessibility, utilization, and cost-effectiveness play an important role in expanding development around the world^1,2^. Agricultural residues and non-nutritional biomasses have a significant potential for energy generation after coal, natural gas and oil^3–6^. Therefore, bioprocesses can deliver multiple energy-like products and strengthen the circular bio-economy^7,8^. Biofuels address environmental issues, fuel security, and socioeconomic benefits to ease the reliance on fossil fuels for sustainable development^9^. Bio-oil obtained from lignocellulosic biomass is expected to play a significant role in future fuel generation as it possesses a higher energy density than biomass^10–12^. For the sustainability of biofuel production, bioenergy is connected to the idea of a holistic circular economy worldwide. Bioprocesses involve processing, selecting, and converting feedstock into value-added yields such as bioenergy and chemicals. Elliot^13^ revealed that the chemicals obtained from the bio-energy process are used in various applications such as textile industries, plastics, paints, automobiles, and food additives. Biomass largely refers to forest and agricultural residues, the most favorable feedstocks for their heating value, low cost, and availability^14^. Biomass residues can be transformed into energy carriers using biochemical or thermochemical conversion routes^15–17^.

**Figure 1:**
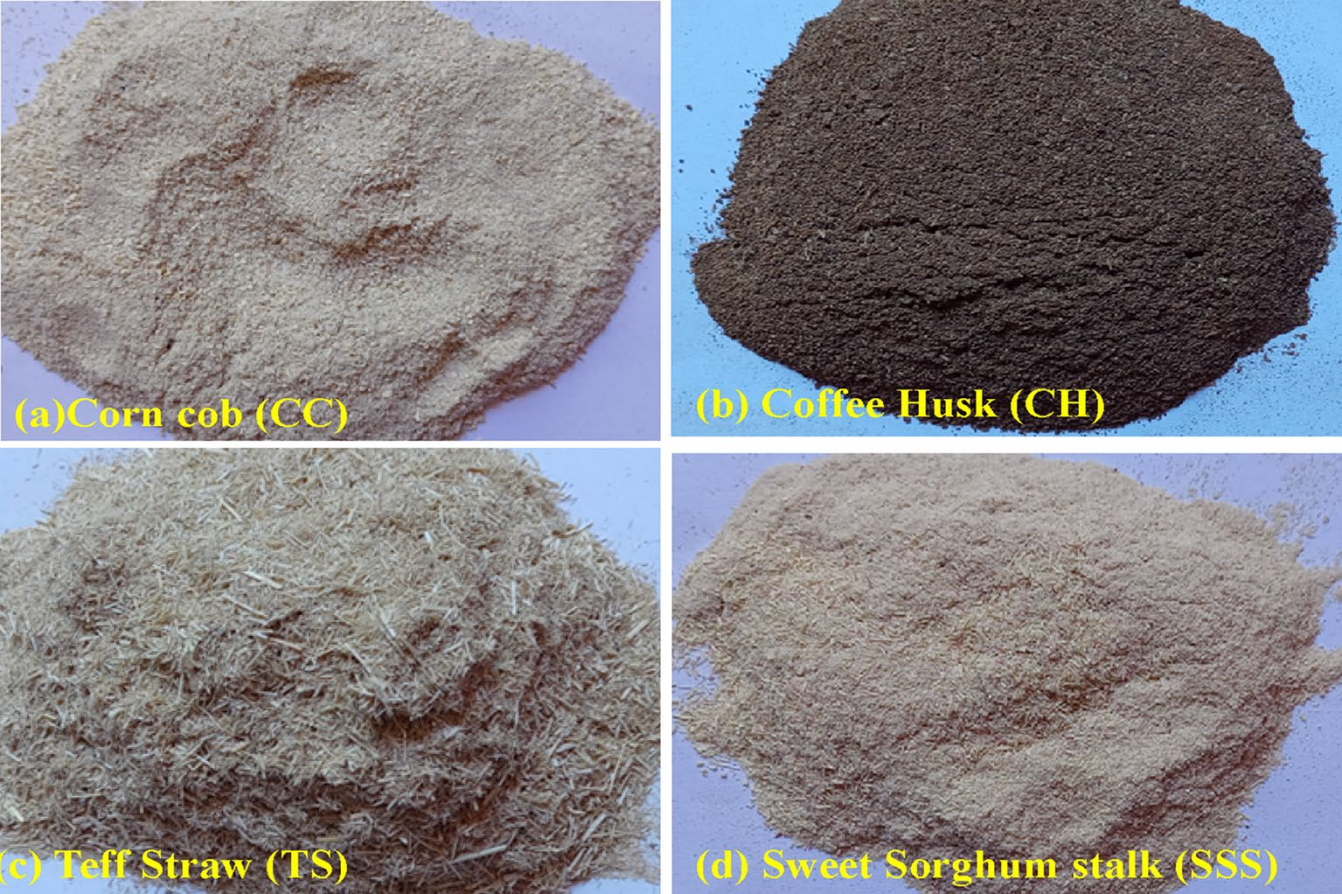
Four types of biomass samples are (**a**) CC, (**b**) CH, (**c**) TS, (**d**) SSS.

Teff straw (TS), coffee husk (CH), corn cob (CC), and sweet sorghum stalk (SSS) residues are agricultural wastes obtained from teff, corn, sweet sorghum, and coffee processing, as shown in Fig. 1. Teff [Eragrostis tef (Zuccagni.) Trotter] straw, locally known as ch'ed, is derived from teff grain, mainly found in Eritrea, Northern Kenya, and Ethiopia^18^. It comprises 25% teff grains and 75% teff straw, with an annual production of 4.3 million and 13 million tonnes, respectively^19,20^. Teff straw is considered a nutritious fodder for ruminants compared to other cereals^21,22^.

Coffee husk is a byproduct of coffee processing in Ethiopia, accounting for 36.8% of the total coffee production, equivalent to 1,68,249.6 metric tonnes in 2021/22^23–25^. Currently, coffee husks have no profitable uses, and their accumulation will lead to a major environmental crisis. Sweet sorghum, known as Tinkish, is traditionally rich in sugar concentration and consumed as feed and food without processing. Sorghum is the third most significant crop in terms of overall production at 50, 243, 68,072 kg (15.70%) with 1.83 million hectares (14.13%) of the area as per CSA (2018) data^26^. Sorghum stalks are utilized as fencing materials for industries, construction, and animal nutrition^26–28^. It is also used for ethanol production due to its drought and salt tolerance^29^. The corn cob is the remaining central part of maize after removing corn kernels. Corn is a significant beverage and food production crop, with a total annual production of 24 million metric tonnes, resulting in a substantial accumulation of unutilized corn cobs^30,31^.

This study focuses on the physicochemical and thermal characterization of four specific biomass residues, namely teff straw (TS), coffee husk (CH), corn cob (CC), and sweet sorghum stalk (SSS), found in Ethiopia. There was a lack of literature on the detailed characterization of these specific biomass types. The study fills an important knowledge gap by comprehensively investigating these biomass residues' physicochemical and thermal properties. It provides valuable insights into these biomass resources' potential utilization and conversion. Another aspect of novelty and importance is that this study appears to be the first to explore the physicochemical and thermal characterization of agricultural biomass residues in Ethiopia. Therefore, the study contributes to understanding Ethiopia's biomass potential, opening avenues for sustainable resource management, renewable energy generation, and waste valorization.

Therefore, the present work is focused on the proximate analysis, ultimate analysis, heating values and compositional analysis of CC, CH, TS and SSS residues commonly available as agriculture waste products in Ethiopia. Further, CC, CH, TS and SSS residues were characterized by X-ray diffraction (XRD), Energy Dispersive X-ray, scanning electron microscopy (SEM), thermogravimetric and differential analysis in an oxidized and inert atmosphere at four (10, 50, 75 and 100 °C min^−1^) heating rates and FTIR.

## Methodology

Biomass of four types (CC, CH, TS and SSS) was received from the local agricultural land (Kombolcha, Ethiopia) and dried for 5 days under 37% humidity at a temperature of 23 °C. The biomass samples were then crushed by motor and piston and passed through standard screens to obtain a particle size of < 300 µm. The samples were packed in air-tight containers and stored for further characterization. CC, CH, TS and SSS samples were composed of lignin, cellulose, hemicellulose, and a small fraction of an extractive, and their compositional analysis was accomplished as per the technique studied by Mudasir et al.^32^. The proximate analyses of the CC CH, TS and SSS samples were examined as per ASTM standard procedures. All obtained values for moisture, fixed carbon, ash and volatile matter were moisture-free.

### Proximate and ultimate analyses

The moisture contents of the TS, CC, CH and SSS samples were obtained per ASTM standard using the E871-82 (2013) procedures^33^. The standard size < 300 µm fine powder samples were taken in the crucible separately and placed inside the hot air oven. The temperature was maintained at 100 ± 5 °C for 1 h, and samples were weighed at regular intervals until a constant weight was obtained. Further, all samples were cooled in desiccators to room temperature. The ash content of the samples was determined using the ASTM standard procedures using the D1102-84 (2013) procedure^34^. 1 g of the oven-dried biomass samples was retained in a weighed crucible for 2 h at a temperature of 750 °C. Biomass samples were removed cautiously and weighed separately.

Volatile matter of the CC, CH, TS and SSS samples was measured based on the E872-82 (2013) procedure^35^.1 g of finely ground biomass up to < 300 µm was kept inside the furnace at 600 °C and raised to 950 °C for 6 min in a closed crucible to obtain volatile matter (VM). The fixed carbon of residues was obtained by subtracting the percentage of moisture, VM and ash from 100. The remaining residue represents the fixed carbon content.

The ultimate analysis percentage of carbon, hydrogen, oxygen, nitrogen, sulfur and ash CC, CH, TS and SSS samples was accomplished using a Vario MICRO superuser CHNS elemental analyzer per ASTM procedure D5373-08^36^. Higher heating values of the CC, CH, TS and SSS samples were obtained by automatic bomb calorimeter (Scientech, Delhi, India) using ASTM procedure D2015-85^37^ with a water equivalent of 2579. Thermogravimetric (TG) and differential (DTG) analysis were conducted using a TG/DTA thermogravimetric analyzer (Perkin Elmer, USA) under both inert and oxidizing atmospheres. Four distinct heating rates (10, 50, 75 and 100 °C min^−1^) were used in non-isothermally degrading studies within 40 to 900 °C. Nitrogen was utilized as the carrier gas, maintaining a constant low rate of 200 mL min^−1^. Throughout the TG/DTG analysis, open platinum sample pans were used to hold the samples. The weight loss data of all biomass samples, along with temperature and time information, were employed in conjunction with the Ozawa–Flynn–Wall (OFW) and Kissinger–Akahira–Sunose (KAS) models to determine the activation energy (*E*a), the reaction model f ($\upbeta$) and correlation factor (R) by using TG/DTG data. A conversion value of 0.2–0.8 was used in this investigation.

The kinetic parameters like correlation factor, reaction model f(α) and activation energy were estimated using Kissinger–Akahira–Sunose and Ozawa–Flynn–Wall iso-conversational models. The thermal conversion of CC, CS, TS and SSS resulted in solid, liquid and gaseous fuels in the work described below.

The decomposition of different biomass can be expressed as:

$${\text{Different}}\;{\text{Biomass}} \to {\text{Bio - oil}} + {\text{Bio - char}} + {\text{Gas}}$$

1 $$k=A{e}^{-{E}_{a}/RT}$$

Ea, k, T, R and A are activation energy, rate constant, temperature (K), universal gas constant (8.314 J/K.mol) and pre-exponential, respectively. Biomass decomposition is commonly described as:

2 $$\frac{d\mathrm{\alpha }}{dt}=K\left(T\right)f\left(\upbeta \right)$$

where f(β) is the kinetic reaction model, α is the conversion rate, and K(T) is the rate constant at temperature T. Rate of thermal conversion of biomass is a function of reactant conversion at a constant temperature, which can be estimated as follows:

3 $$\alpha = (Mo - Mt)/((Mo - Mf)$$

where M_o_, M_t_ and M_f_ are the initial mass, the mass of the sample and the final mass after time t, respectively. On combining Eqs. (1) and (2), therefore, Eq. (2) can be modified as follows:

4 $$\frac{d\mathrm{\alpha }}{dt}=A{e}^{-\left(\mathrm{Ea }/RT\right)}f\left(\upbeta \right)$$

For dynamic cases, the temperature is dependent on both time and heating rates and is described as follows:

5 $$\frac{d\mathrm{\alpha }}{dT}= \frac{A}{\upbeta }{e}^{-\left(\mathrm{Ea }/RT\right)}f\left(\upbeta \right)$$

where (β = dT/dt) after integration and rearranging, Kissinger–Akahira–Sunose (KAS) formula was developed and expressed as follows:

6 $${\text{Ln }}\left( {\beta /{\text{T}}^{{2}}_{{\text{m}}} } \right) = {\text{ln }}\left[ {{\text{AE}}_{{\text{a}}} /{\text{R}}_{{\text{f}}} } \right] - {\text{E}}_{{\text{a}}} /{\text{RT}}_{{\text{m}}}$$

KAS method is used to calculate the activation energy on a plot of ln (β/T^2^_m_) versus 1/T, and the slope is − Ea/R.

The activation energy was also estimated by Flynn Wall Ozawa (FWO) model as follows:

7 $$\log (\beta ) = \log [{\text{AEa}}/{\text{Rf}}] - 2.315 - 0.457{\text{Ea}}/{\text{RTm}}$$

Plot, log (β) versus 1/T for four heating rates (10, 50, 75, and 100 °C min^−1^) parallel lines and for conversion as from 0.2 to 0.8 values corresponding to activation energy is obtained from the slope of 0.457 Ea/R.

### SEM, EDX and X-ray diffraction

The morphology of the CC, CH, TS and SSS samples was revealed using SEM (SEM 500, Carl Zeiss Microscopy GmbH, Germany). Images were taken with a system and gun vacuum of 7.12 × 10^−6^ and 2.29 × 10^−9^ mbar at 10 kV with 1000 magnifications.

The XRD analysis of the biomass residues was conducted using the Smart Lab X-ray diffractometer (Rigaku Corporation, Smart Lab 3 kW, Japan). For the powder diffraction, 1 g of each biomass sample was utilized. The XRD measurements were performed using a 2.2 kW Cu–K anode X-ray source (40 kV, 30 mA), covering an angular range of 1.5 h (10–90).

The FTIR measurements were conducted using the Avatar 370 instrument from Shimadzu, Bruker: OptikGmbh, USA. The sample powder was mixed with 1% potassium bromide (KBr) to prepare the samples for analysis. Spectra were recorded within the range of 500–4000 cm^−1^, with a resolution of 4 cm^−1^. A mathematical correction was applied to the generated spectrum to account for ATR (Attenuated Total Reflection).

## Results and discussion

### Compositional analysis

Determining the composition of specific biomass is vital to producing final bio-products during thermal conversion processes. Chemical composition plays a significant role in these processes, highlighting the importance of understanding the composition of biomass samples. The values obtained for cellulose, hemicelluloses and lignin of CC, CH, TS and SSS are shown in Table 1 and are comparable within the literature^38–41^.

**Table 1:** Compositional Analysis of Raw CC, CH, TS and SSS and its Comparison.

Raw material	Lignocellulosic composition (wt%, daf)			References
	Cellulose	Hemicellulose	Lignin	
CC	41.15	32.02	20.11	Present work
CH	33.25	23.9	25.07	
TS	31.56	29.80	22.67	
SSS	36.42	30.1	19.85	
CC	39	35	15	38
CH	47.29		27.14	39
TS	41.8	38	17	40
SSS	39.5	29.8	22.2	41

*CC* Corn cob, *CH* Coffee Husk, *TS* Teff Straw, *SSS* Sweet Sorghum stalk.

### Proximate and ultimate analysis

Proximate analysis is crucial to investigating the thermo-chemical conversion processes of the CC, CH, TS and SSS residues. The experiments show that CC, CH, TS, and SSS are characterized by < 10% moisture content^32^. The highest and lowest moisture content are in TS (8.76) and SSS (6.40), respectively. The high moisture content affects the heating values, while the low moisture content helps in conversion processes for fuel production^39^. The ash content is within the acceptable range of the CC, CH, TS and SSS residues, providing a huge benefit during furnace design. The highest and lowest percentages of ash content obtained in TS (7.23%) and CC (2.79) residues are low when correlated with other biomasses^39,42^. The favorable suitability of CC, CH, TS, and SSS residues for thermochemical conversion processes is attributed to their low ash content.

Conversely, high ash concentrations are undesirable as they can negatively impact the burning rate and lead to reactor corrosion, incrustation, and slag formation^39,42^. Inorganic elements such as Fe, Ca, Mg, P, and K are important in conversion. Alkali metals with low melting points contribute to operational issues like fouling, corrosion, and slag formation^43^. The volatile matter content is crucial to understanding combustion mechanisms and designing and operating fuel conversion systems. Among the biomass residues, SSS exhibited the highest volatile material percentage (71.71%), while CC had the lowest (56.74%)^39^. Balasundram et al.^44^ further emphasized that biomass residues with higher volatile contents contribute to the formation of bioenergy in the form of oil and gas. The obtained fixed carbon content is within the range of 14.77–31.83% revealing the generation of solid-like products. The moisture, ash, volatile matter and fixed carbon content of the CC, CH, TS and SSS are comparable to those of other biomasses such as walnut shells, coffee husk, wheat straw, and wood pellets, as shown in Table 2.

**Table 2:** Proximate Analysis of Raw CC, CH, TS and SSS and its Comparison.

Biomass type	Proximate analysis result (%w/w)				VM/FC	References
	Moisture	Ash	V. Matter	Fixed Carbon		
CC	8.64	2.79	56.74	31.83	1.78	Present work
CH	8.45	6.20	68.60	16.75	4.10	
TS	8.76	7.23	69.23	14.77	4.69	
SSS	6.40	3.94	71.71	17.95	4.00	
CC	5.1	8.5	65.1	21.3	–	46
CH	7.92	3.54	71.63	16.9	–	24
TS	7.3	4	74.7	14.0	–	47
SSS	5.98	2.89	89.85	–	–	48
WS	8.06	0.33	76.38	15.23	–	32
CH	9.06	3.55	77.09	19.36	–	39
WS	8.31	9.38	75.19	15.43	–	49
WP	6.43	1.26	81.57	17.17	–	49

*CC* Corn cob, *CH* Coffee Husk, *TS* Teff Straw, *SSS* Sweet Sorghum stalk, *WS* Walnut shell, *CH* Coffee husk, *WS* Wheat Straw, *WP* Wood pellets.

The major elements of CC, CH, TS and SSS biomass residues are hydrogen, carbon, sulfur and nitrogen. Biomass residues generally have a high oxygen and hydrogen percentage and a low carbon, sulfur and nitrogen percentage compared to coal^45^. During thermal conversion processes, carbon and hydrogen are vital energy sources, and the obtained values were similar to those of other biomass residues^24,46–50^. Low and high percentages of carbon and oxygen decrease the higher heating value of lignocellulosic wastes^51^. Consequently, fuels with high carbon and less oxygen percentage are attractive for energy uses^52^. The low percentage values of *H/C* and *O/C* ratios dipped the emissions of vapors and gases^53^. The amount of nitrogen and sulfur in the biomass is low, contributing to the declining NO_x_ and SO_x_ production^54^. The higher heating values of the CC, CH, TS and SSS residues are within the range of 17.3–19.7 MJ/kg, comparable to biomass, as shown in Table 3^55^.

**Table 3:** Comparative analysis of elemental composition and high heating values of raw CC, CH, TS, and SSS biomass residues.

Biomass types	Ultimate Analysis (wt %)							References
	C	H	N	S	C/H	C/N	HHV (MJ/kg)	
CC	44.4	5.9	0.52	0.01	7.49	86.16	17.6	Present work
CH	40.78	5.69	1.44	0.01	7.17	28.35	19.7	
TS	40.99	5.58	0.83	0.07	7.34	49.62	17.3	
SSS	44.03	5.68	0.29	0.07	7.75	151.2	19.3	
CC	47.26	5.79	0.56	0.5	8.16	84.39	18.15	53
CH	46.83	4.81	0.44	0.05	9.73	106.43	22.7	24
TS	41.9	6.5	0.7	0.3	6.44	59.85	NA	47
SSS	50.74	6.71	0.61	0.08	7.56	83.18	22.8	48

*CC* Corn cob, *CH* Coffee Husk, *TS* Teff Straw, *SSS* Sweet Sorghum stalk.

### SEM and energy dispersive X-ray spectra analysis

**Figure 2:**
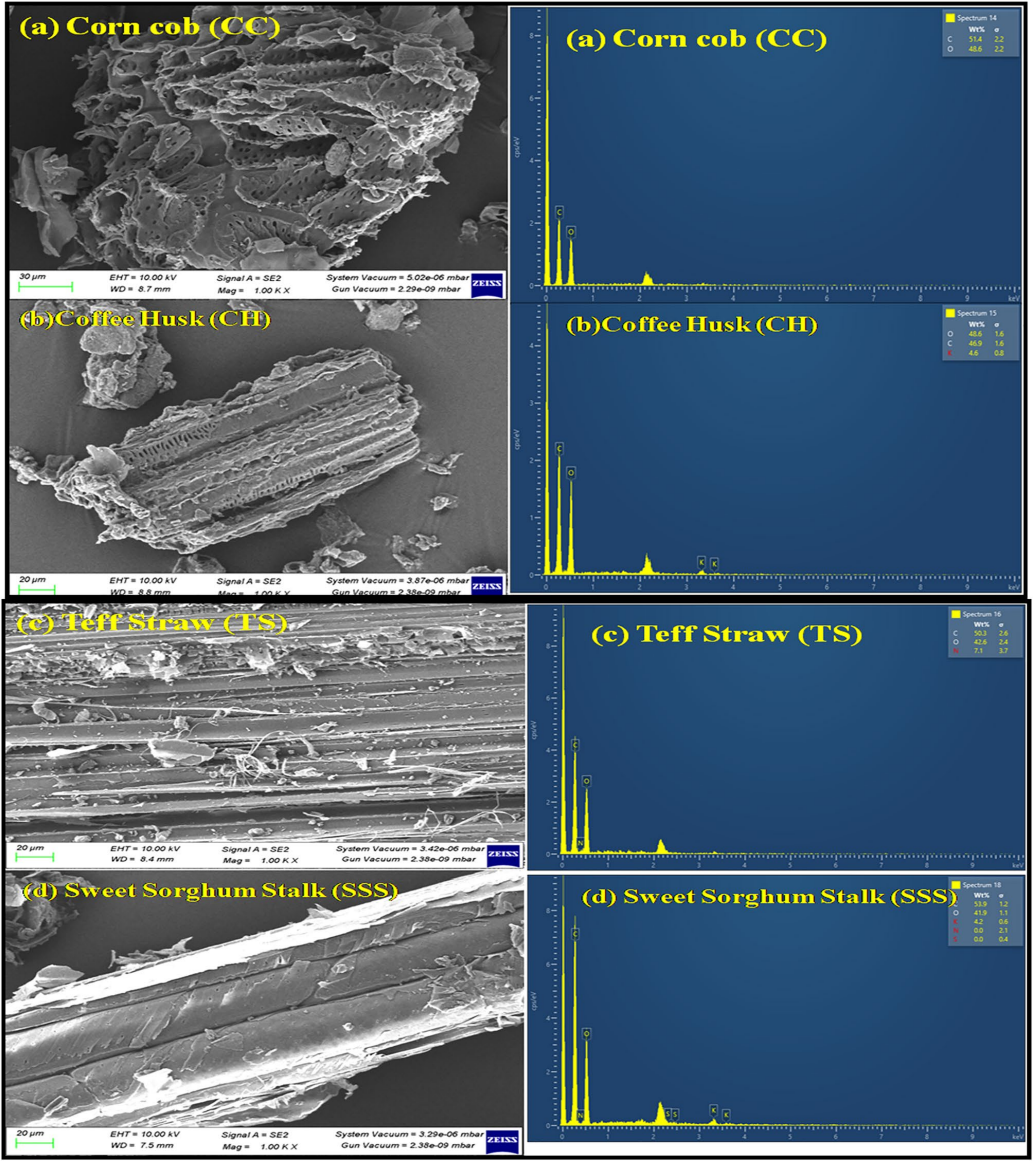
SEM and EDX Spectra of the (**a**) CC (**b**) CH (**c**) TS (**d**) SSS with magnification 1000.

The SEM images were combined with the results of EDX characterizations of CC, CH, TS, and SSS biomass residues to examine their surface morphology, as revealed in Fig. 2. The SEM analysis indicated that the structures of the biomass residues are irregular and agglomerated, displaying a rough texture. Notably, the samples did not exhibit pores but showed abundant parallel lines, which serve as nutrient and water transportation pathways from the soil. While CC and TS exhibited a rocky structure, CH and SSS displayed a sheet-like, planar structure. The SEM micrographs also revealed the presence of unburnt ash residues in the CC, CH, TS, and SSS samples, suggesting incomplete conversion processes^56–59^.

The EDX analysis revealing the main composition of carbon and oxygen, along with small amounts of nitrogen (in TS) and potassium (in CH and SSS), is shown in Fig. 2. The EDX spectra exhibit prominent peaks for carbon and oxygen and minor peaks for nitrogen and potassium, as indicated by the weight percentages in Table 4. The abundance of carbonaceous material suggests its significant potential as a precursor for various thermochemical conversion processes such as gasification, pyrolysis, and activated carbon production^50^. Moreover, the presence of potassium in CH and SSS residues may contribute to the formation of sticky surfaces, leading to additional agglomeration^60,61^.

**Table 4:** EDX analysis results of CC, CH, TS and SSS.

Biomass samples	Elements (wt. %)			
	C	O	N	K
CC	51.43	48.57	0	0
CH	45.30	47.44	2.87	4.39
TS	50.28	42.63	7.09	0
SSS	53.90	41.87	0	4.23

### X-ray diffraction (XRD)

**Figure 3:**
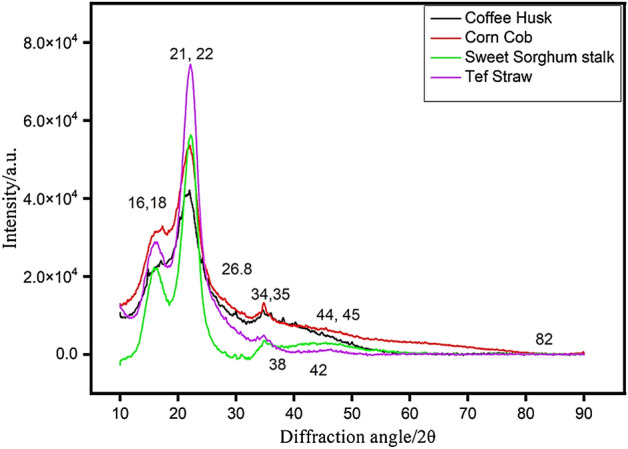
XRD of the CC, CH, TS, and SSS biomass samples.

The XRD patterns of CC, CH, TS, and SSS samples are scanned in the range of 2*θ* = 10 − 90° and are presented in Fig. 3.The diffractograms displayed for biomass samples possess high-intensity peaks at 2*θ* values of 21°, 22°, 16° and 18° and low intensity at 2*θ* values of 26.8°, 34°, 35°, 38°, 42°, 44° and 45°. These extensive peaks at 16°, 18°, 21°, and 22° revealed that biomass samples possess high carbon contents that are crystalline and can be seen in all biomass samples. Among all these biomass samples, the maximum crystallinity was obtained for TS and the minimum for CH. These are the findings derived from the current study. The obtained values were comparable with the results reported by Tesfaw^40^, for teff straw, Lateef for corncob^62^, Gabriel^63^ for teff straw, coffee hull, sugarcane bagasse and inset fiber, Rambo^64^ for coffee husks, açai seed, rice husks, bamboo, coconut husks and banana stalks, Xu^65^ for corn stover, corn cob and sorghum stalk.

### Fourier transform infrared spectroscopy (FTIR)

**Figure 4:**
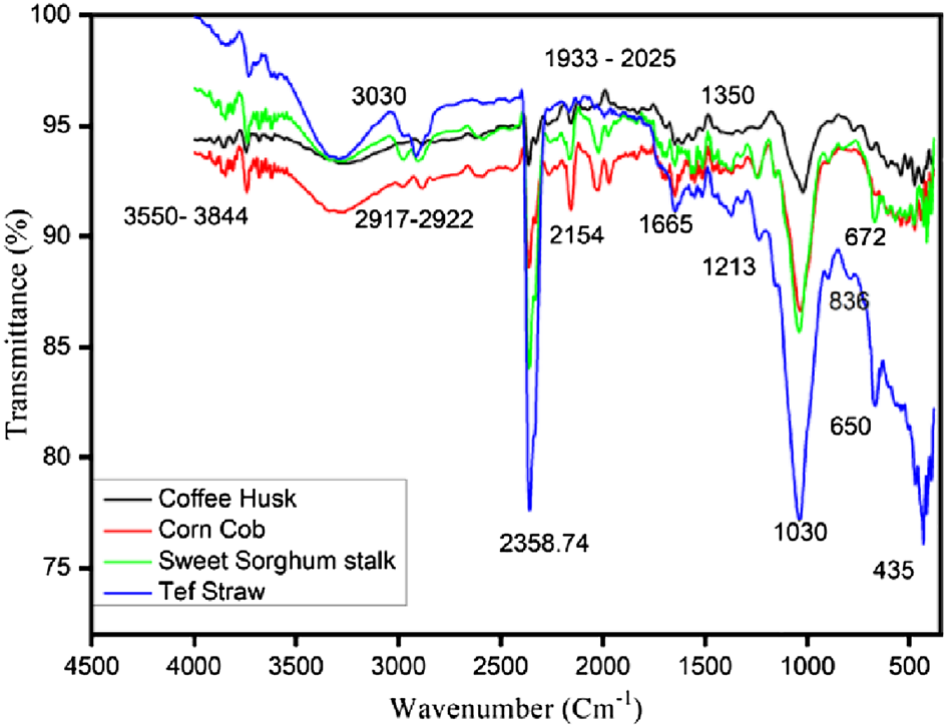
Infrared spectra of the CC, CH, TS, and SSS biomass samples.

The FTIR spectra of the CC, CH, TS, and SSS were recorded for direct information about functional groups at wavelengths in the 4500 to 400 cm^−1^ range, as shown in Fig. 4 in Table 5. The result shows that the regions 3550–3844 cm^−1^ and 2917–2922 cm^−1^ indicated the –OH stretching vibration in hydroxyl groups and the –CH (CH_2_ and CH_3_) asymmetric and symmetric stretching vibrations, respectively. While the region 1030–1213 cm^−1^ confirms the –CO stretch. Very intense peaks in the 1665–1350 cm^−1^ region originate from the stretching mode of carbonyls, mainly ketones and esters*.* The peaks near 600 cm^−1^ and 1030 cm^−1^depict C–O stretching and C–H bending, respectively. These vibrations originated in cellulose, hemicelluloses, and lignin. The chemically active bonds in functional groups accelerate the thermal conversion reactions of biomass^66^. During thermal decomposition, the –OH group will accelerate the rate of condensation reactions led by dihydroxylation of the cellulose, while C–H existence leads to hemicellulose degradation^66^. The existence of the alkenes (C=C) group leads to lignin decomposition, while the carboxylic groups (C–O) group in hemicellulose and cellulose accelerate the rate of decarboxylation reactions that result in the breakage of glycosidic bonds and form a series of oxygen-containing compounds such as aldehydes, acids, ethers and non-condensable gases such as CO_2_and CO^66–70^.

**Table 5:** FTIR Spectra Band Assignments of Raw CC, CH, TS and SSS.

Band assignment	Band frequency (cm^−1^)			
	CC	CH	TS	SSS
O–H stretching	3742	3744	–	–
C–H stretching	3679,3620	–	–	3679, 3622
Stretching vibrations of C–C/C=C in the aromatic structure	–	–	2914	2906
C–O–H aromatic skeletal vibrations in-plane bending	2364	2364	2358	2361
CH deformation and OH bending vibrations	2154, 1969	2156	–	2163
Stretching vibrations of C–C and C–O in theguaiacyl unit in lignin	1514	1647	–	1514
C–OH stretch and C–H in-plane deformation in syringyl	1246	–	–	1243
C–OH and O–CH_3_ stretching	1033	–	1039	1041
C–H out-of-plane stretching	473, 446 432, 416	543, 507, 473, 437	472, 432,	432, 414
C–H out-of-plane stretching	392	393	392	–

### Thermal decomposition analysis in an oxidized and inert atmosphere

**Figure 5:**
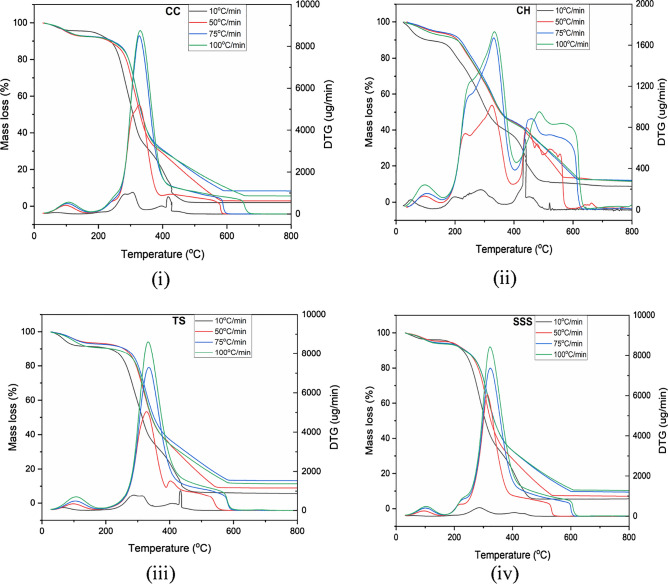
TGA and DTG investigation of CC, CH, TS and SSS in an oxidizing atmosphere at four different heating ranges.

**Figure 6:**
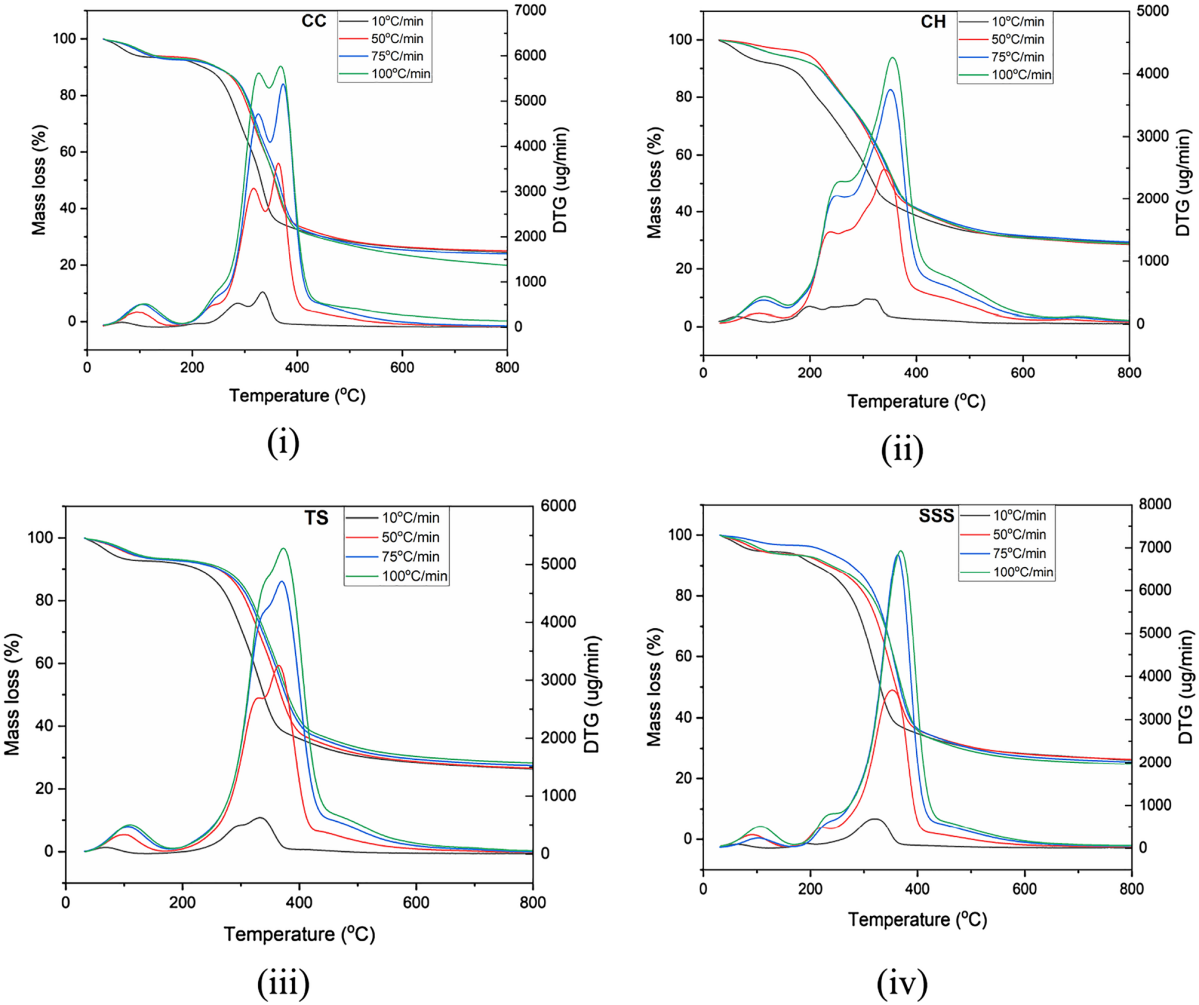
TGA and DTGanalysis of (**i**) CC, (**ii**) CH, (**iii**) TS, and (**iv**) SSS in an inert atmosphere at four different heating ranges.

Thermogravimetric (TG) and differential thermogravimetric (DTG) mass loss curves of CC, CH, TS and SSS were obtained experimentally in an oxidized and inert atmosphere at four different heating rates (10, 50, 75, and 100 °C min^−1^) are presented in Figs. 5 and 6i–iv. Dehydration, devolatilization and carbonization are the three main stages of the thermal degradation process of the CC, CH, TS and SSS residues to describe their thermal behavior^71–73^.In the first thermal curve, 6–9% mass loss is observed due to the loss of low molecular volatiles and intrinsic or extrinsic bonded water vapors for the temperature range between 37 and 120 °C. The maximum and lowest mass losses in the thermal degradation curve were observed in TS residues (≈ 9%) and SSS residues (≈ 6.40%) at a heating rate of 10 and 50 °C/min, respectively, as shown in Fig. 5. The second zone ranging from 120 to 380 °C exhibited significant mass loss (40–85%) in all biomass wastes, with variations depending on the heating rate, 45–76% at 10 °C min^−1^, 50–85% at 50, 75 and 100 °C min^−1^. This stage corresponds to the devolatilization/oxidation of hemicelluloses, cellulose and lignin^71,72,74^. The third zone, the passive zone, begins at 390 °C and extends to 900 °C, where there is minimal mass loss due to carbonaceous solids and lignin degradation over a wide temperature range^72,75^. Throughout the investigation, it was observed that thermal degradation above 450 °C was nearly insignificant. The displacement of the TG mass loss curves at higher heating rates can be attributed to variations in heat flux within the CC, CH, TS, and SSS biomass particles. Lower heating rates facilitate better heat transfer, leading to more uniform biomass degradation^76^. The thermal decomposition of CC, CH, TS, and SSS occurred over a broad temperature range, with overlapping degradation of constituents, as depicted in Fig. 5i–iv.

The derivative curve at the lowest heating rate (10 °C min^−1^) provides a clearer depiction of cellulose, hemicelluloses, and lignin degradation. Notably, a significant mass loss occurs in the temperature range of 325–550 °C for all samples, primarily due to the degradation of cellulose and hemicelluloses, with lignin contributing to a smaller extent^76–81^. Therefore, in the active thermochemical conversion zone, major and minor reactions can be attributed to the decomposition of hemicelluloses and cellulose. This finding aligns with similar observations reported by other researchers studying biomass degradation in this temperature range^75,82,83^. The thermal decomposition of CC, CH, TS, and SSS occurs over a wide temperature range, with the degradation of hemicellulose and lignin components overlapping, as illustrated in Figs. 5 and 6i–iv. The derivative curve of mass loss at the lowest heating rate (10 °C/min) reveals significant degradation of the lignocellulosic material, characterized by merged peaks as the heating rate increases. Cellulose and hemicelluloses exhibit significant degradation from 120 to 390 °C, while lignin undergoes partial degradation within this temperature range^77,78^. The DTG curves become larger and wider with increasing heating rates from 10 to 100 °C/min. This indicates simultaneous decomposition of the lignocellulosic biomass components, resulting in overlapping curves due to their heterogeneous composition^84^. Higher heating rates reduce the time required to reach the pyrolysis temperature, resulting in a shorter pyrolysis time, which is considered beneficial^85,86^.

### Activation energy determination

**Figure 7:**
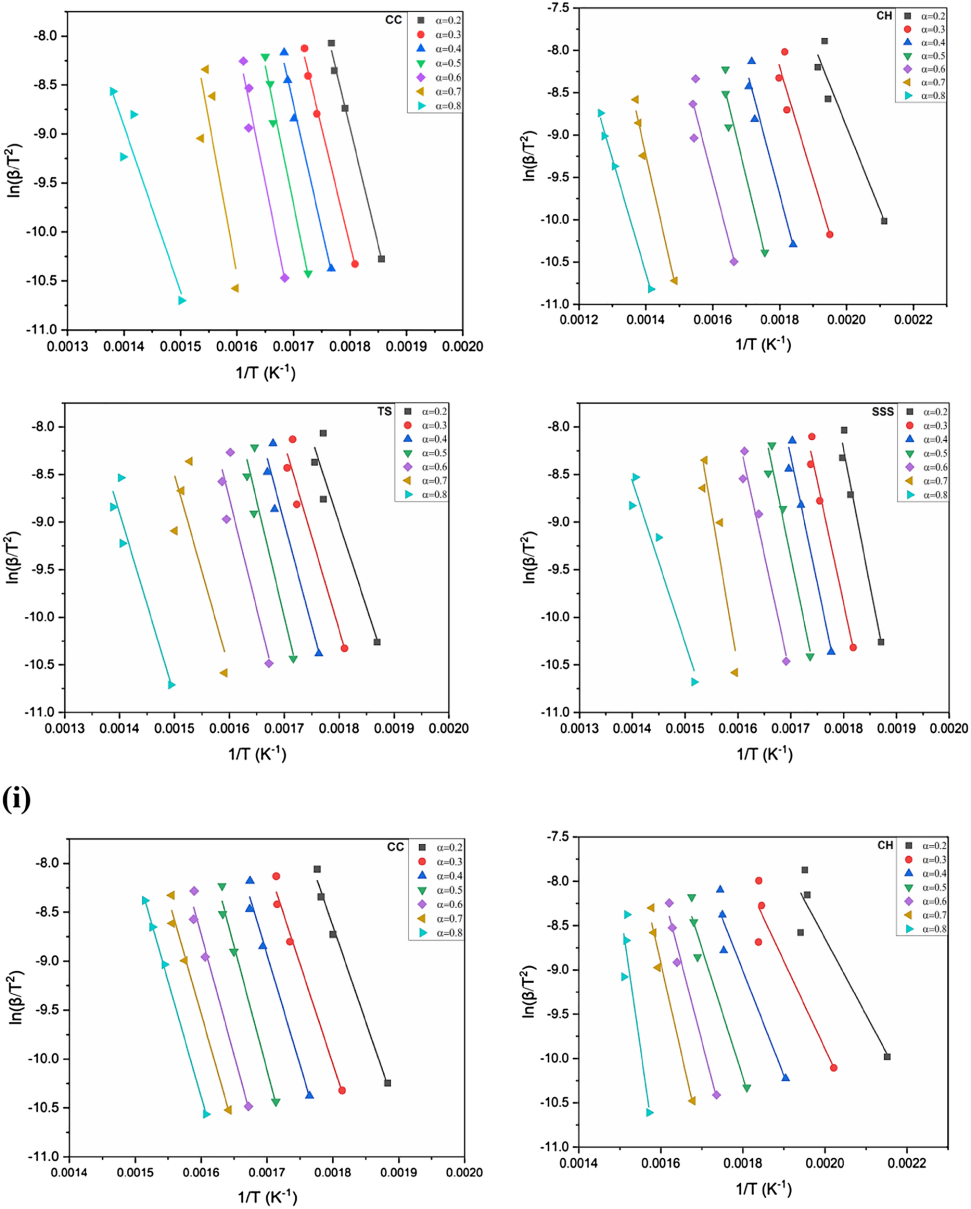

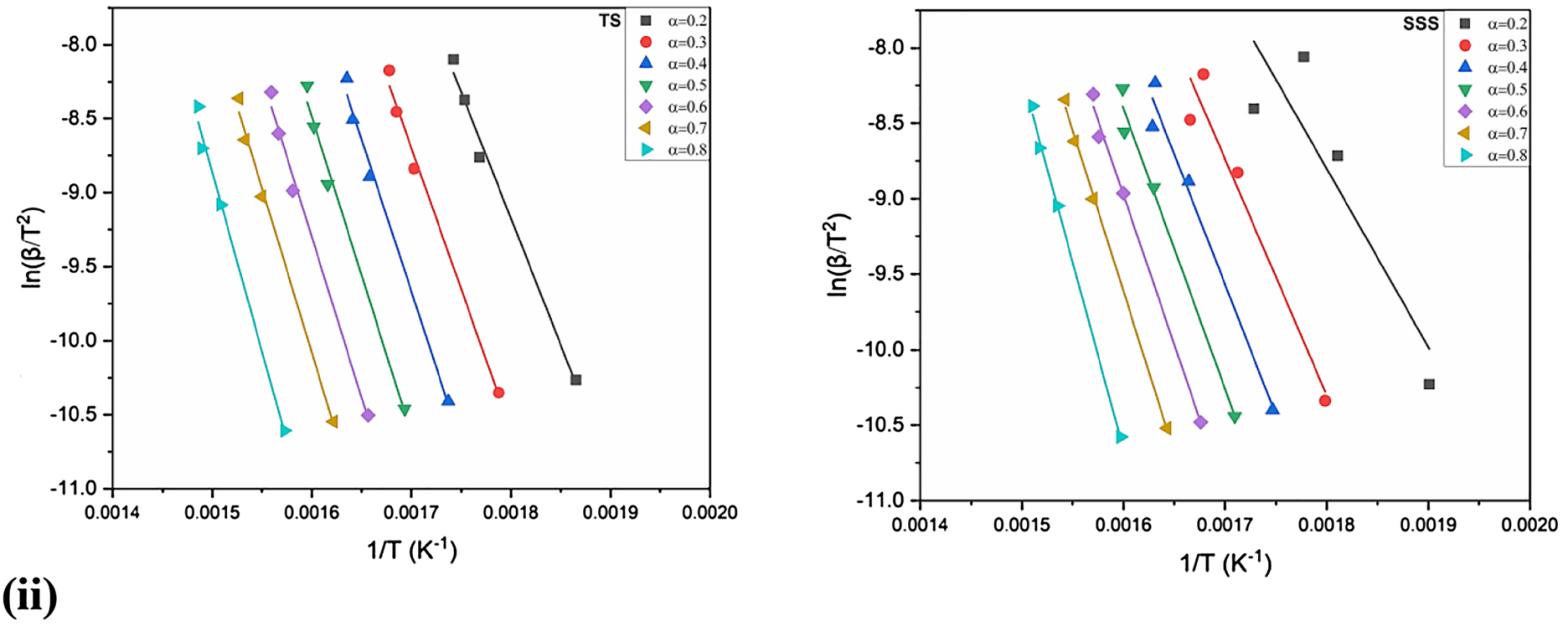
(**i**) Arrhenius plots of ln(β/(T)^2^) versus 1/(T) of CC, CH, TS and SSS in an oxidizing atmosphere with conversion rate (0.2–0.8) obtained by the KAS method. (**ii**) Arrhenius plots of ln(β/(T)^2^) versus 1/(T) of (i) CC (ii) CH (iii) TS and (iv) SSS in an inert atmosphere obtained by KAS with a conversion rate (0.2–0.8).

**Figure 8:**
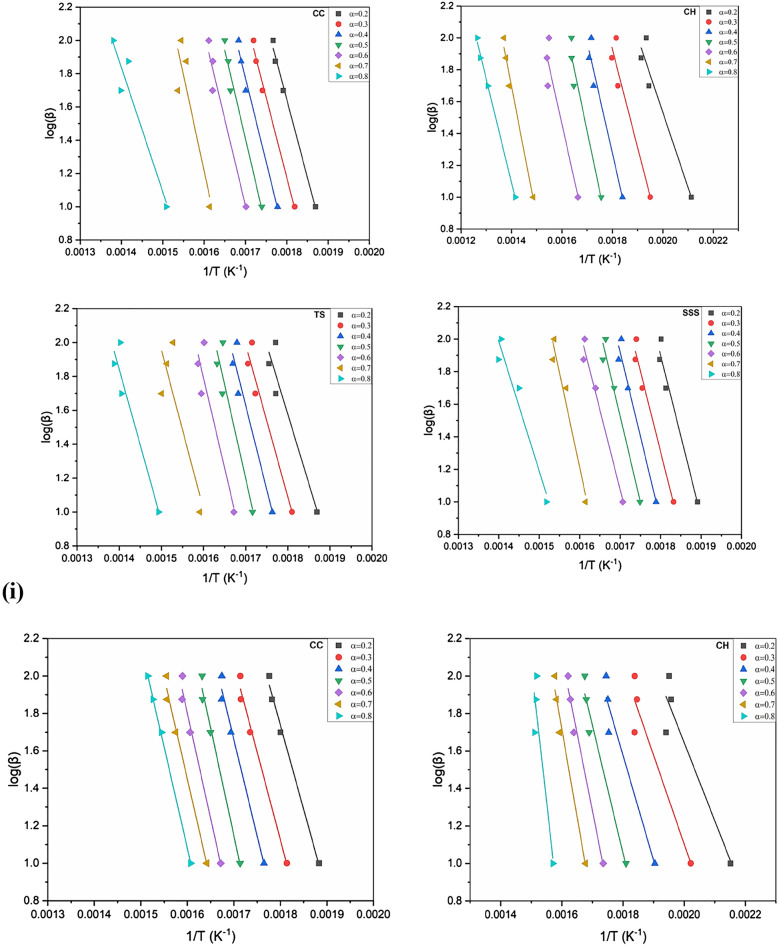

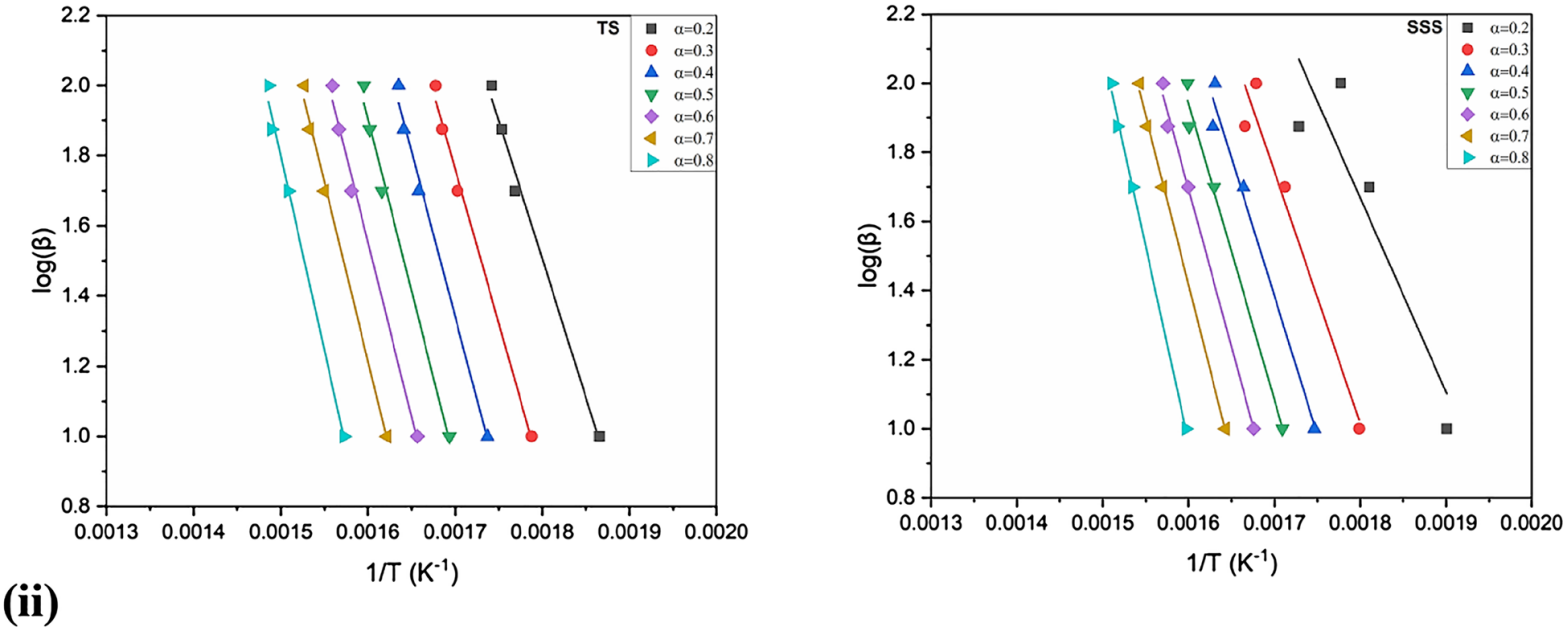
(**i**) Arrhenius plots of log(β/) versus 1/(T) of CC, CH, TS and SSSobtained by OFW method in an oxidizing atmosphere with conversion rate (0.2–0.8). (**ii**) Arrhenius plots of log(β/) versus 1/(T) of CC, CH, TS and SSS in an inert atmosphere obtained by OFW method with conversion rate (0.2–0.8).

**Figure 9:**
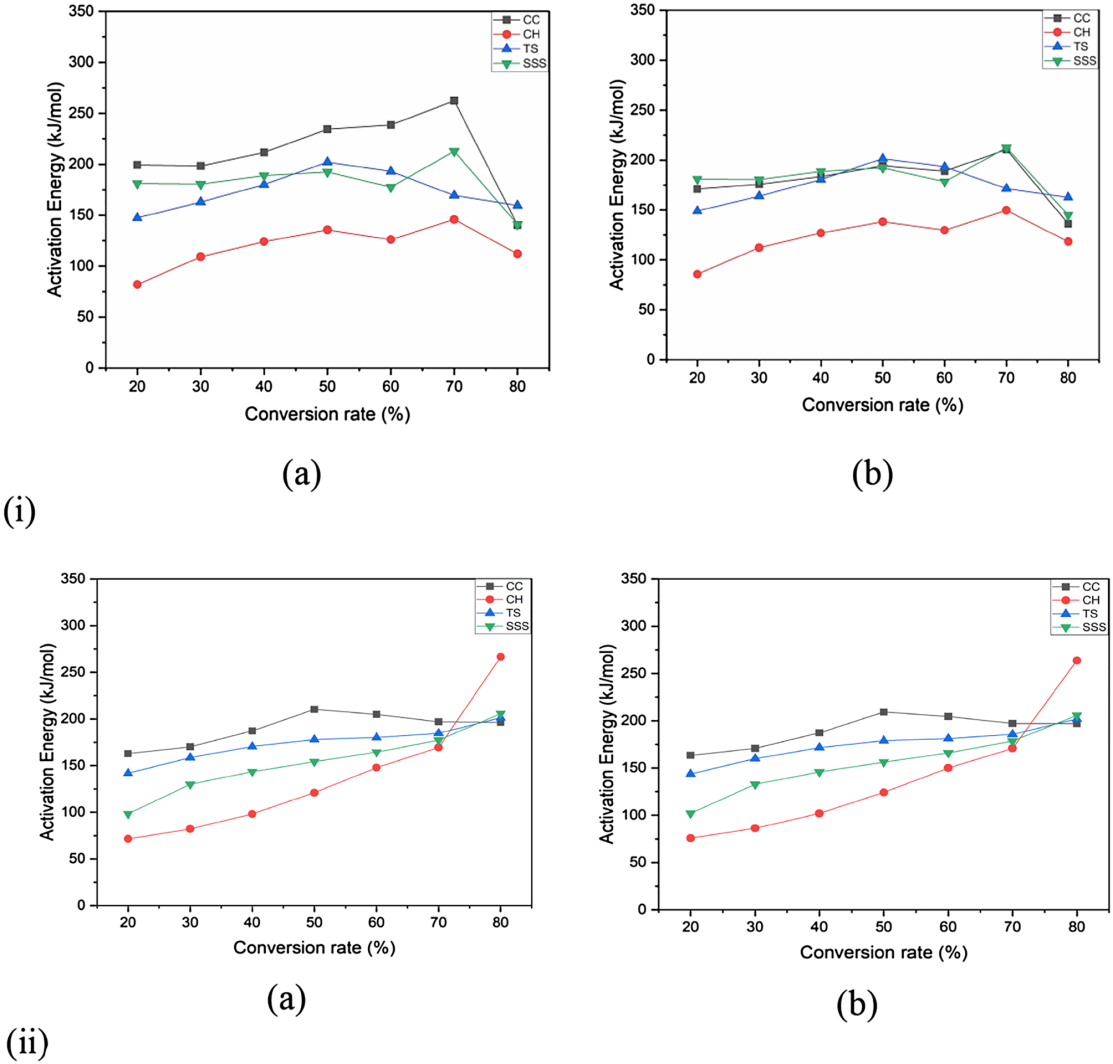
(**i**) Variations in activation energy with a CC, CH, TS and SSS conversion ratefor KAS and OFW method in anoxidized atmosphere. (**ii**). Variations in activation energy with CC, CH, TS and SSS conversion rate for KAS and OFW method in an inert atmosphere.

The behavior of the activation energy and correlation coefficients (R^2^) in the OFW and KAS methods was generated from the linear regressions at four different heating rates as a function of the conversion (0.2–0.8) in the inert and oxidizing atmospheres exposed in Figs. 7, 8 and 9i, ii. The correlation coefficients (R^2^) range from 0.74 to 0.99 in CC, 0.89–0.99 in CH, 0.85–0.93 in TS and 0.93–0.97 in SSS in the KAS method. Similarly, the OFW method ranges from 0.80 to 0.99, 0.90–0.99, 0.72–0.94 and 0.94–0.97 in CC, CH, TS and SSS, respectively. It was revealed that the pre-exponential values of CC, CH, TS and SSS samples are almost the same and increase with increased heating rates.

The average activation energy (E_a_) values generated in all biomass samples by the KAS model are slightly higher than those of the OFW model. The results revealed that the maximum and minimum E_a_ for the KAS model were 262.238 and 81.919 kJ mol^−1^atconversions of 0.7 and 0.2 of CC and CH, respectively. The OFW model has 12.349 (0.7) and 85.737 kJ mol^−1^ (0.2) SSS and CH, respectively. Activation energy with conversion doesn’t show any regular arrangement due to removing light volatile and moisture contents. However, the average E_a_is very close, signifying that the KAS and FWO models are appropriate for finding this kinetic parameter. The lowest E_a_ is more feasible for a pyrolysis reaction. The conversions of CC, CH, TS and SSS were nearly parallel, increasing the chances of a single reaction mechanism. Thus, the rise of E_*a*_ may be due to the influence of heat transfer at higher temperatures in both models^87^. Ma et al.^88^ investigated deviations in the E_a_due to various competitive and parallel reactions during biomass degradation. Primarily, the obtained E_*a*_ values were low due to the cracking of the weaker bonds in hemicellulose. The comparisons of activation energies in the present work are comparable with other different biomasses presented in the literature^39,89–93^.

## Conclusions

In this research, a comprehensive investigation was conducted to examine the physical- and thermo-chemical characteristics along with FTIR, SEM, EDX and XRD of four different biomass residues (TS, CH, CC and SSS). Thermal degradation showed complete moisture removal at 37–120 °C in the first zone. The second zone ranging from 120 to 380 °C was attributed to the devolatization/oxidation of cellulose and hemicelluloses, resulting in significant mass loss (40–85%). The third zone spanning 390–900 °C indicated the degradation of carbonaceous solids and lignin with minimal mass loss. During pyrolysis, lignin and cellulose/hemicellulose thermal stability were evident, with different temperature ranges for their decomposition influenced by the heating rate. KAS and FWO models could efficiently predict the Ea of any conversion process. The high fixed carbon and volatile matter content and low ash and moisture content suggested that CC, CH, TS, and SSS residues hold potential as viable sources for biofuel generation through gasification or pyrolysis. The SEM showed a heterogeneous structure, while the EDX and XRD spectra showed a high carbonaceous material and a crystalline structure. FTIR analysis highlighted the dominance of oxygenated compounds in the composition of CC, CH, TS, and SSS residues. This comprehensive characterization confirms that biomass samples are excellent energy sources for various thermo-chemical conversion processes.

## Future work

The CC, CH, TS, and SSS residue utilization can focus on the following areas to further enhance sustainable energy production and value-added products:

1. Explore novel pyrolysis and gasification technologies that can enhance the efficiency of CC, CH, TS, and SSS residue conversion and increase the yield of biofuels and value-added chemical products.
2. The by-products obtained during the thermal conversion processes identify potential high-value applications in different industries such as fuel, energy, agriculture, construction, or wastewater treatment.
3. The feasibility of establishing integrated biorefineries that can efficiently utilize multiple biomass residues to produce a range of products, including biofuels, biochemicals, and bio-based materials.
4. Develop and apply advanced kinetic models that account for the complex interactions between various components of biomass residues during thermal degradation. Integrating computational techniques and experimental data will help accurately predict reaction pathways and kinetics, improving process optimization.
5. Conducting a comprehensive techno-economic analysis to evaluate the economic viability of scaling up the conversion processes for commercial applications. This would consider the feedstock cost, process efficiency, and potential revenue streams from different products.
6. Focus on developing environmentally sustainable biomass conversion processes that minimize greenhouse gas emissions and promote a circular economy. Investigate carbon capture and utilization technologies to offset emissions and enhance the overall carbon sequestration potential.
7. Exploring innovative technologies to upgrade the quality and properties of bio-oil and syngas produced during conversion.

By undertaking these research initiatives, we can unlock the full potential of biomass residues as renewable energy sources and valuable feedstocks for a wide range of bio-based products. This effort may contribute to a more sustainable, environmentally friendly, diversified energy and chemical landscape.

## Data Availability

The datasets used and analyzed during the current study are available from the corresponding author upon reasonable request.

## Abbreviations

TS: Teff straw
CH: Coffee husk
CC: Corn cob
SSS: Sweet sorghum stalk
KAS: Ozawa–Flynn–Wall
FWO: Kissinger–Akahira–Sunose
SEM: Scanning electron microscopy
EDX: Energy dispersive X-ray
XRD: X-ray diffraction
FTIR: Fourier transform infrared spectroscopy
TGA: Thermogravimetric
DTG: Differential thermogravimetric
K: Potassium
P: Phosphorus
Mg: Magnesium
Ca: Calcium
Fe: Iron
O: Oxygen
H: Hydrogen
T: Temperature
ASTM: American Society for Testing Materials
NOx: Oxides of nitrogen
SOx: Oxides of sulphur
C: Carbon
S: Sulfur
R: Correlation coefficients
VM: Volatile matters
FC: Fixed carbon
N: Nitrogen
KBr: Potassium bromide
ATR: Attenuated total reflection
SDG: Sustainable energy goal

## List of symbols

MJ/kg: Mega Joules per kilogram
kJ mol^−^^1^: Kilo Joules per mole
°C min^−^^1^: Celsius per minute
wt%: Weight percentage
daf: Dry ash free
cm: Centimeter
°C: Celsius
Ea: Activation energy
A: Pre-exponential
k: Rate constant
R: Universal gas constant
f(β): Reaction kinetic model
α: Conversion rate
K(T): Rate constant at temperature
Mo,: Initial mass
Mt: Mass of sample after time t
Mf: Final mass after time t
